# Feminizing *Wolbachia*: a transcriptomics approach with insights on the immune response genes in *Armadillidium vulgare*

**DOI:** 10.1186/1471-2180-12-S1-S1

**Published:** 2012-01-18

**Authors:** Frédéric Chevalier, Juline Herbinière-Gaboreau, Delphine Charif, Guillaume Mitta, Frédéric Gavory, Patrick Wincker, Pierre Grève, Christine Braquart-Varnier, Didier Bouchon

**Affiliations:** 1 Université de Poitiers, Laboratoire Écologie, Évolution, Symbiose, UMR CNRS 6556, 40 avenue du recteur Pineau, F-86022 Poitiers cedex, France; 2 Université de Lyon 1, Laboratoire de Biométrie et de Biologie Évolutive, UMR CNRS 5558, 43 boulevard du 11 novembre 1918, F-69622 Villeurbanne, France; 3 Parasitologie Fonctionnelle et Évolutive, UMR CNRS 5244, Université de Perpignan, 52 avenue Paul Alduy, 66860 Perpignan cedex, France; 4 Génoscope, UMR CNRS 8030, Centre National de Séquençage, Evry, France

## Abstract

**Background:**

*Wolbachia* are vertically transmitted bacteria known to be the most widespread endosymbiont in arthropods. They induce various alterations of the reproduction of their host, including feminization of genetic males in isopod crustaceans. In the pill bug *Armadillidium vulgare*, the presence of *Wolbachia* is also associated with detrimental effects on host fertility and lifespan. Deleterious effects have been demonstrated on hemocyte density, phenoloxidase activity, and natural hemolymph septicemia, suggesting that infected individuals could have defective immune capacities. Since nothing is known about the molecular mechanisms involved in *Wolbachia*-*A. vulgare* interactions and its secondary immunocompetence modulation, we developed a transcriptomics strategy and compared *A. vulgare* gene expression between *Wolbachia*-infected animals (*i.e.*, “symbiotic” animals) and uninfected ones (*i.e.*, “asymbiotic” animals) as well as between animals challenged or not challenged by a pathogenic bacteria.

**Results:**

Since very little genetic data is available on *A. vulgare*, we produced several EST libraries and generated a total of 28 606 ESTs. Analyses of these ESTs revealed that immune processes were over-represented in most experimental conditions (responses to a symbiont and to a pathogen). Considering canonical crustacean immune pathways, these genes encode antimicrobial peptides or are involved in pathogen recognition, detoxification, and autophagy. By RT-qPCR, we demonstrated a general trend towards gene under-expression in symbiotic whole animals and ovaries whereas the same gene set tends to be over-expressed in symbiotic immune tissues.

**Conclusion:**

This study allowed us to generate the first reference transcriptome ever obtained in the Isopoda group and to identify genes involved in the major known crustacean immune pathways encompassing cellular and humoral responses. Expression of immune-related genes revealed a modulation of host immunity when females are infected by *Wolbachia*, including in ovaries, the crucial tissue for the *Wolbachia* route of transmission.

## Background

*Wolbachia* are endosymbiotic α–Proteobacteria that are maternally transmitted and cause various reproductive manipulations in a wide range of invertebrate hosts (see [1] for a review). *Wolbachia* infection is widespread in Crustacea where species of the three main classes (Malacostraca, Ostracoda, and Maxillipoda) were found to be infected [2]. *Wolbachia* prevalence reaches ~60% in terrestrial isopods (order Oniscidea). In the pill bug *Armadillidium vulgare*, one of the most intensively studied examples, *Wolbachia* are responsible for inducing the development of genetic males into functional females. This is achieved by preventing the androgenic gland differentiation responsible for male development [3,4]. Consequently, in the progenies of infected mothers the proportion of females reaches 70 to 80% according to the transmission rate of *Wolbachia *[5,6]. This modification of the host sex ratio leads to a low proportion of males in the field reached 20% as evidenced by a meta-analysis of 57 populations [2]. Since *Wolbachia* vertical transmission is dependent on the reproductive success of their hosts, it could be expected that the infection provides fitness benefit that could promote dispersion of *Wolbachia* in the host population. Surprisingly, most field populations of *A. vulgare* are not infected by *Wolbachia *[2], which could reflect the conflicting relationships between the pill bug and the bacteria. As some life history traits of *A. vulgare* are directly impacted by *Wolbachia*, the low prevalence of the infected specimens in natural populations could be due to various factors that reduce the host fitness. Feminizing *Wolbachia* have the potential to reduce male to female ratio to values limiting mating possibilities and therefore limiting population size [7]. Furthermore, males are able to distinguish between infected and uninfected females [7]. This mating preference could lead to a sexual selection in favor of uninfected females. Rigaud and Moreau [8] also demonstrated that after multiple mating, sperm depletion in males affects fertility only in infected females. In addition, a reduced fertility and survival is recorded in *Wolbachia*-infected females [6,9,10]. However, these females had a higher reproductive investment (they produce more offspring and more eggs per clutch) so ultimately the reproductive success is similar between infected and non-infected females [6]. More recently, deleterious effects have been demonstrated on immunocompetence of infected females [10,11]. Indeed, these females have a lower hemocyte density, a decrease in PO activity, and a more severe hemolymph septicemia that could result in a reduced life span in *A. vulgare *[10,11]. This latter effect could impact host fitness including lower or higher resistance to intruders as it has been shown in many insect species [12]. For example, it has been demonstrated that *Wolbachia* suppress the host defence of *Drosophila simulans* against parasitoids [13]. Conversely, *Wolbachia*-induced stimulation of the host’s innate immune system has been suggested as a mechanism conferring resistance to pathogens. In *D. melanogaster* and *D. simulans*, *Wolbachia* protect their hosts against RNA viral infection [14-16]. This has also been demonstrated in *Aedes aegypti* where the injection of the life-shortening *w*MelPop *Wolbachia* strain provides resistance against the Dengue and the Chikungunya viruses as well as against *Plasmodium gallinaceum* and *Brugia pahangi *[12,17-21]. In parallel, *Wolbachia* were shown to induce immune gene expression in different biological systems. For example, a *Wolbachia*-infected cell line displayed an overexpression of antioxidant proteins that are key components of *Ae. albopictus* immune response [22,23]. Similarly, host immune genes are up-regulated in *Ae. aegypti *[17] and *Anopheles gambiae *[18] when infected by *w*MelPop.

Since nothing is known about the molecular mechanisms involved in *Wolbachia*-*A. vulgare* interactions and its secondary immunocompetence modulation, different Expressed Sequence Tag (EST) libraries [normalized, non-normalized, and Suppression Subtractive Hybridization (SSH) libraries] were constructed in order to generate a large transcriptomics data set. To identify genes involved in *Wolbachia-*host association and in host immune response, EST and SSH libraries were prepared using RNA from ovaries (*i.e.*, the tissue involved in vertical transmission) and from *A. vulgare* females artificially challenged by *Salmonella typhimurium*. Host gene expression in *Wolbachia*-infected individuals was then compared to uninfected individuals by *in silico* and *in vitro* subtractions. This analysis revealed a set of potentially modulated immune genes. Expression of immune genes were investigated to examine whether the decrease of immunocompetence in the *Wolbachia*-infected *A. vulgare* may be related to modulation of the host innate immune system.

## Methods

This work has been conducted in parallel in two other invertebrate models (*i.e.*, *Asobara tabida-Wolbachia* and *Sitophilus oryzae-SPE* (*Sitophilus* primary endosymbiont)) in order to determine conserved and divergent immune pathways and to ascertain whether the invertebrates have selected common strategies to control their symbionts and to discriminate between symbionts and pathogens [24,25].

### Symbiotic association

*Armadillidium vulgare* (Crustacea Isopoda) individuals were sampled from two laboratory lineages whose *Wolbachia*-infection status is known. Animals infected by the feminizing *Wolbachia* strain (*w*VulC) (*i.e.*, “symbiotic” animals) originated from Celles-sur-Belle, France. This lineage has been identified by crossing experiments as composed of all ZZ individuals: ZZ males and ZZ+*Wolbachia* females [2]. Uninfected individuals (*i.e.*, “asymbiotic” animals) with genetic sex determinism (ZZ males and WZ females) originated from Nice, France [2,5,26]. These lines have been stably maintained in the lab since 1967 and 1991 for asymbiotic and symbiotic lineages, respectively. As *A. vulgare* males are never infected by *Wolbachia*, only females (WZ females and ZZ+*Wolbachia* females) were used in this study.

### Bacterial challenge

*Salmonella typhimurium* (strain 12023G) were cultured in LB medium overnight. Dilutions were performed to obtain c10^4^ bacteria.µL^-1^ (OD=0.01). Asymbiotic females were injected with 1 µL of bacterial suspension at the side of sixth pereon segment using a thin glass needle. Females were dissected at 6h, 9h, and 15h post injection. Ovaries, gut, caeca, fat tissues, hemocytes, hematopoietic organ, nerve chain, and brain were conserved in liquid nitrogen separately until total RNA extractions.

### Library constructions

**Figure 1:**
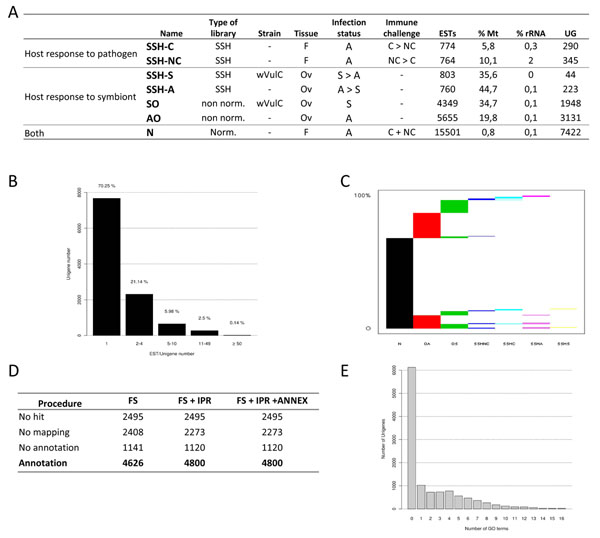
**EST library characteristics** A. Summary of the different EST libraries. Suppression Subtractive Hybridizations (SSHs) were performed with Miror Orientation Selection procedure. cDNA libraries were sequenced with or without normalization (Norm. or Non Norm. respectively). The *w*VulC *Wolbachia* strain (Celles sur Belle, France) induces feminization of genetic males and has some negative impacts in symbiotic females (see text). Immune challenge was performed through the injection of 10^4 ^*Salmonella typhimurium* in asymbiotic females: RNA was extracted 6h, 9h, and 15h after challenge. F = whole female tissues, Ov = ovary tissues, S = symbiotic, A = asymbiotic, C = immune challenge, NC = no immune challenge, ESTs = expressed sequence tags, Mt = mitochondrial genes, rRNA = ribosomal genes, UG = number of unigenes. B. Abundance classes of ESTs and unigenes. C. Unigenes occurrences among EST libraries. The horizontal axe represents the different EST libraries, the vertical axe represents the occurrence of unigenes within the libraries. Horizontal reading of the graph indicates the percentage of unigenes shared by several libraries. D. GO annotation results for High Scoring Pairs (HSP) coverage of 0%. GO annotation was first conducted using the Score Function (SF) of the BLAST2GO software. The GO terms selected by the annotation step were then merged with InterProScan predictions (SF + IPR). Finally, the Annex annotation was run (SF + IPR + ANNEX). E. Annotation distribution of GO terms.

Seven different EST libraries were prepared from different tissues of *A. vulgare* (Figure 1A). Total RNA was extracted as described in [27] and treated with DNAse (TurboDNase, Ambio, Applied Biosystems), following the manufacturer’s instructions.

Two non-normalized libraries were constructed from asymbiotic and symbiotic ovaries (AO and SO) starting with 1 µg of polyA RNAs. They were prepared using Creator SMART cDNA Library Construction kit (Clontech/BD Biosciences), following the manufacturer’s instructions. cDNA was digested by *Sfi*I, purified (BD Chroma Spin – 400 column) and ligated into pDNRlib vector for *Escherichia coli* transformation. Amplified double strand cDNA (ds cDNA) was prepared using a SMART approach [28]. SMART Oligo II oligonucleotide (Clontech/BD Biosciences) and CDS primer were used for first-strand cDNA synthesis. SMART-amplified cDNA samples were further digested by *Rsa*I endonuclease.

The SSH libraries from asymbiotic and symbiotic ovaries (SSH-A and SSH-S) were constructed starting with 20 µg of total RNA. SSH libraries from specimens challenged and not challenged by *S. typhimurium* (SSH-C and SSH-NC) were performed on 20.4 µg of a total RNA equally pooled from different tissues (*i.e.*, ovaries, gut, cæca, fat tissues, hemocytes, hematopoietic organ, nerve chain, and brain) harvested at each time point. The pooled total RNA was obtained by mixing equal amounts of total RNA extracted separately for each tissue and for each time point. Subtractive hybridizations were performed using SSH method in both directions (Asymbiotic *vs.* Symbiotic A/S and vice-versa S/A; Not Challenged *vs.* Challenged NC/C and vice-versa C/NC) as described in [29,30] using the PCR-Select cDNA Subtraction Kit (Clontech/BD Biosciences). SSH libraries were prepared by Evrogen (Moscow, Russia). The Mirror Orientation Selection (MOS) procedure was used for SSH-A/S and SSH-C/NC as described in [31] in order to reduce the number of false-positive clones in the SSH-generated libraries. Purified cDNAs from SSH-A/S and SSH-C/NC were cloned into the pAL16 vector (Evrogen) and used for *E. coli* transformation.

Finally, the normalized library (N) was prepared with 75 µg of a pooled total RNA from an equimolar proportion of asymbiotic and symbiotic ovaries, and 6h, 9h, and 15h challenged asymbiotic females. As for the libraries of challenged specimens, total RNA was extracted separately from the same tissues. This N library was prepared by Evrogen (Moscow, Russia). Total RNA sample was used for ds cDNA synthesis using SMART approach [28]. SMART prepared amplified cDNA was then normalized using Duplex Specific Nuclease (DSN) normalization method [32]. Normalization included cDNA denaturation/reassociation, treatment by DSN [33] and amplification of normalized fraction by PCR. Normalized cDNA was purified using QIAquick PCR Purification Kit (QIAGEN), digested with *Sfi*I, purified (BD Chroma Spin - 1000 column) and ligated into pAL 17.3 vector (Evrogen) for *E. coli* transformation.

### EST sequencing and data processing

**Figure 2:**
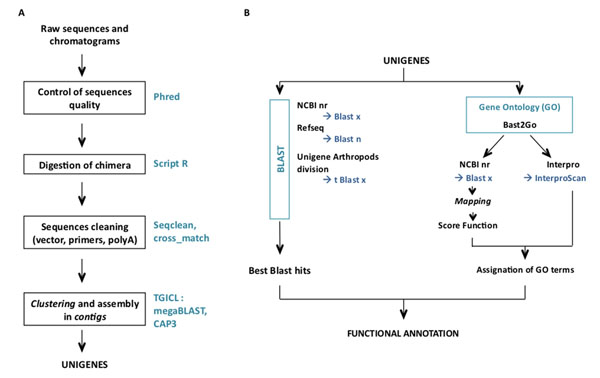
Sequence treatment (A) and functional annotation procedure (B).

All clones from the libraries were sequenced using the Sanger method (Genoscope, Evry, France) and were deposited in the EMBL database [EMBL: FQ884936 to FQ908260]. A general overview of the EST sequence data processing is given in Figure 2. Raw sequences and trace files were processed with Phred software [34] in order to remove low quality sequences (score < 20). Sequence trimming, which includes polyA tails/vector/adapter removal, was performed by cross match. Chimerical sequences were computationally digested into independent ESTs. Clustering and assembly of the ESTs were performed with TGICL [35] to obtain unique transcripts (unigenes) composed of contiguous ESTs (contigs) and unique ESTs (singletons). For that purpose, a pairwise comparison was first performed by a modified version of megaBLAST (minimum similarity 94%). Clustering was done with tclust that proceeds by a transitive approach (minimum overlap: 60bp at 20bp maximum of the end of the sequence). Assembly was done with CAP3 (minimum similarity 94%).

To detect unigene similarities with other species, several BLASTs (with a high cut-off e-values) were performed against the following databases: NCBI nr [BLASTx (release: 1 March 2011); e-value < 5, HSP length > 33aa], Refseq genomic database (BLASTn, e-value < 10), Unigene division Arthropods (tBLASTx, #8 *Ae. aegypti*, #37 *An. gambiae*, #3 *Apis mellifera*, #3 *Bombyx mori*, #53 *D. melanogaster*, #9 *Tribolium castaneum*; e-value < 5), and *Wolbachia* sequences from Genbank (Release 164; e-value < 1e^-20^). Gene Ontology (GO) annotation was carried out using BLAST2GO software [36]. In the first step (mapping), a pool of candidate GO terms was obtained for each unigene by retrieving GO terms associated to the hits obtained after a BLASTx search against NCBI nr. In the second step (annotation), reliable GO terms were selected from the pool of candidate GO terms by applying the Score Function of BLAST2GO with “permissive annotation” parameters (EC-weight=1, e-value-filter=0.1, GO-weight=5, HSP/hit coverage cut-off =0%). In the third step of the annotation procedure, the pool of GO terms selected during the annotation step was merged with GO terms associated to InterPro domain (InterProScan predictions based on the longest ORF). Finally, the Annex augmentation step was run to modulate the annotation by adding GO terms coming from implicit relationships between GO terms [37].

### Statistical analyses on libraries

We used the randomization procedure (with 500 random datasets) and *R* statistics described in [38] to detect unigenes whose transcript abundance (number of ESTs) was statistically different in AO and SO libraries (at a false discovery rate of 2.5 %). In order to extract biological processes and molecular functions statistically over-represented in SO libraries, we performed a hyper-geometrical test between GO terms from the SO library and those from the AO library, which represents the natural physiological conditions. The p-values were then adjusted using Bonferroni’s correction.

In order to perform a functional enrichment analysis of the unigenes extracted from the SSH, we used the FatiGO web tool [39] against the SO library. With respect to the GO analysis, four different levels of description (3, 4, 6, and 9) were chosen for the biological processes.

### Quantitative expression by Real-Time RT-PCR

Gene expression quantification was performed in whole animal, ovaries, and immune tissues (hemocytes and hematopoietic organs pooled) of asymbiotic and symbiotic females.

#### RNA extractions

For the whole animal condition, each individual was crushed with pestle and mortar in liquid nitrogen. Total RNA extraction was performed from about 30 mg of powder with TRIzol® reagent according to the manufacturer's instructions (Invitrogen). For ovaries and immune tissues, total RNA extractions were performed from 25 and 50 females respectively with RNeasy Mini Kit according to the manufacturer's instructions (QIAGEN).

#### Real-Time RT-PCR

First-strand cDNA was synthesized with the SuperScript III kit (Invitrogen) in accordance with manufacturer’s instructions, starting from 1 µg of total RNA using random hexamer primers. For whole animal samples, 0.2 µg of 5 individual extractions were pooled in 1 µg. Three biological replicates of each sample (whole animals, ovaries, and immune tissues) were used.

For each gene, primer pairs were designed with the Real-time PCR function of PerlPrimer [40]. The Tm and the length of each primer pair were fixed at 60°C and 18-22 bp, respectively. Primers used for quantitative PCR are summarized in Additional File 1.

Quantitative RT-PCR was performed using LightCycler LC480 system (Roche) as follows: 10 min at 95°C, 45 times [10 sec at 95°C, 10 sec at 60°C, 20 sec at 72°C]. A melting curve (65°C to 97°C) was recorded at the end of each reaction in order to check that the PCR product was unique. The reaction mixture consisted of 1.25 µL of each primer (10 µM), 5 µL of Fast SYBR-Green Master Mix (Roche) and 2.5 µL of diluted cDNA (corresponding to 12.5 ng of cDNA). Standard curves were plotted using 4 dilutions (125 ng, 25 ng, 5 ng, 1.25 ng) of pooled cDNAs from whole animals and ovaries. Efficiency of the PCR reaction was calculated.

Expression data for each gene were estimated using the efficiency of the primer pair and the crossing point [41]. All gene expressions were normalized by the geometric mean of the expression level of the L8-ribosomal (RbL8) and Elongation Factor 2 (EF2) reference genes. Normalization and statistical pair-wise comparisons have been determined using REST [42].

## Results

### First reference transcriptome in isopods

ESTs were generated from seven high quality cDNA libraries, including four SSH libraries, two non-normalized libraries and one normalized library. Characteristics of cDNA libraries are summarized in Figure 1A. A total of 28 606 ESTs (mean length: 504 ± 170 bp) were generated which covered around 14.4 Mb. Clustering of all EST sequences was performed by TGICL [35] resulted in 10 923 unique transcripts (*i.e.*, unigenes which covered 6.4 Mb). About 75% of the clusters contained one EST (*i.e.*, singletons; n = 8 211) and 25% contained ESTs assembled in a consensus sequence (*i.e.*, contigs, n = 2 712). The normalized library and the ovary libraries contained a greater proportion of contigs which is likely due to the deeper sequencing of these libraries (Figure 1C.). The average length of these unigenes was 590 ± 250 bp with a GC content of 33.5% and an average coverage of 3.5 (Figure 1B)

Functional annotation was performed on all 10 923 unigenes through BLASTx and tBLASTx similarity searches against various databases. Because of the ancient divergence between *A. vulgare* and the closest sequenced genomes we used a cut-off threshold of 1e^-05^. A total of 44% of the unigenes had BLAST similarities to known sequences, mainly from *Ae. aegypti* (10.5%), *An. gambiae* (8.7%), *D. melanogaster* (7%), and different malacostracans (3.1%) with an e-value lower than 1e^-20^ for 64.8% of the unigenes. The remaining 66% of unigenes showing no match could correspond to species-specific genes or UTR extremities of the cDNA.

### Functional analysis

GO annotation was carried out using BLAST2GO software (Figures 1D, 2B). A total of 42% of unigenes were annotated after the BLAST2GO annotation procedure for High Scoring Pair (HSP) coverage of 0%. While we kept the unigenes/GO dataset corresponding to the minimum HSP coverage percentage, the mean number of GO terms assigned per unigene was low (1.18 GO term/unigene, Figure 1E).

To determine the effect of *Wolbachia* on host gene expression, an *in silico* subtraction was performed between libraries of symbiotic (SO) and asymbiotic (AO) ovaries. In these libraries, a total of 4564 unigenes have been annotated and based on the *R* statistics, only 6 unigenes were differentially represented: 3 unigenes were over-represented in symbiotic ovaries while 3 were over-represented in asymbiotic ovaries. Unfortunately, these unigenes could not be identified by BLAST and only one is associated to a biological function (see Additional File 2: Unigenes differentially represented between symbiotic and asymbiotic ovaries). The immune processes were over-represented in symbiotic ovaries (Table 1 and Additional File 3: Processes and functions over-represented in *A. vulgare* ovaries in response to *Wolbachia* infection, biological process levels 4 and 6). Indeed, 21 and 15 unigenes with immune gene similarities were identified in AO and SO libraries, respectively (Additional File 4: Immune unigenes present in SO, AO, SSH-S, SSH-A, SSH-C, and SSH-NC libraries).

**Table 1:** Functions over-represented in *A. vulgare* ovaries in response to *Wolbachia* infection.

	Biological process	GO accession	A	S	A/S
**AO ~ SO**	cell fate determination	GO:0001709	0.02	0.05	**0.40**
**level 3**	immune effector process	GO:0002252	0.07	0.16	**0.44**
**(n= 99)**	regulation of immune system process	GO:0002682	0.04	0.14	**0.29**
	generation of a signal involved in cell-cell signaling	GO:0003001	0.04	0.05	**0.80**
	muscle contraction	GO:0006936	0.02	0.07	**0.29**
	chromosome segregation	GO:0007059	0.18	0.23	**0.78**
	ensheathment of neurons	GO:0007272	0.00	0.02	**0.00**
	circadian rhythm	GO:0007623	0.07	0.09	**0.78**
	cell recognition	GO:0008037	0.02	0.07	**0.29**
	reproductive behavior	GO:0019098	0.04	0.05	**0.80**
	membrane docking	GO:0022406	0.04	0.05	**0.80**
	viral reproductive process	GO:0022415	0.02	0.05	**0.40**
	cellular pigmentation	GO:0033059	0.04	0.05	**0.80**
	leukocyte activation	GO:0045321	0.05	0.09	**0.56**
	regulation of response to stimulus	GO:0048583	0.12	0.18	**0.67**
	coagulation	GO:0050817	0.09	0.11	**0.82**
	regulation of body fluid levels	GO:0050878	0.04	0.05	**0.80**
	endocrine process	GO:0050886	0.11	0.14	**0.79**
	cellular response to stimulus	GO:0051716	0.05	0.07	**0.71**

In the same manner, two *in vitro* SSHs were performed by subtracting common transcripts between symbiotic and asymbiotic ovaries (SSH-S), and reciprocally (SSH-A). These SSHs were contaminated by a high proportion of mitochondrial ESTs (~40%) that were removed for further analyses. To reveal the functions over-represented, we compared each SSH to SO library by the FatiGO web tool. One biological process (vesicle transport along microtubule) and one molecular function (microtubule motor activity) were over-represented in asymbiotic ovaries (Table 2). Most of the 223 unigenes that are associated to these two GO terms belong to the kinesin-like protein family. In these two libraries, the BLAST analyses allowed the identification of 1 immune gene in SSH-S and 6 immune genes in SSH-A libraries respectively (Additional File 4: Immune unigenes present in SO, AO, SSH-S, SSH-A, SSH-C, and SSH-NC libraries).

**Table 2:** Functional enrichment analysis: list of GO terms that were over-represented in the lists of unigenes obtained by SSH experiments on ovaries (FatiGO web tool). P-value of Fisher's exact unilateral tests. Adjusted p-value for multiple test correction.

Test	# unigenes	Ontology domain	Level	Term	GO ID	p-value	Adj. p-value
SSH-A versus SO	223	Biological process	9	vesicle transport along microtubule	GO:0047496	1.35E-04	5.97E-02
		Molecular function	3	microtubule motor activity	GO:0003777	1.13E-03	9.85E-02
							
SSH-S versus SO	44			*no significant term*			

In order to identify genes expressed in response to pathogenic bacteria, we performed SSH libraries between *S. typhimurium*-challenged and unchallenged asymbiotic *A. vulgare* females (SSH-C) and reciprocally (SSH-NC). We thus identified 31 and 29 unigenes in SSH-C and SSH-NC libraries, respectively, that are related to crustacean immune processes. In the SSH-C library these immune related unigenes exhibited a greater diversity than those of the SSH-NC library (Additional File 4: Immune unigenes present in SO, AO, SSH-S, SSH-A, SSH-C, and SSH-NC libraries).

Finally, 30 non redundant immune related unigenes were identified in libraries constructed from symbiotic/asymbiotic conditions (SO/AO, SSH-S/SSH-A) and 59 in libraries constructed from challenged/not challenged conditions (SSH-C/SSH-NC) (Additional File 3: Processes and functions over-represented in *A. vulgare* ovaries in response to *Wolbachia* infection, biological process levels 4 and 6). Among them, 28 unigenes were successfully amplified by PCR. In addition, 16 other unigenes were selected from the normalized library (N) for their putative involvement in major immune processes. Annotations were further confirmed by protein domain identification (CD Search vs the Conserved Domain Database on NCBI server [43]). If the complete domain pattern of a given protein was not found, the suffix “-like” was added to the unigene name (Table 3). Expression of these 44 genes were further analysed by RT-qPCR.

**Table 3:** List of immune genes identified in the libraries.

												Library	occurrences			
	Biological function	Gene	BLAST program	Accession	Description	Species	e-value	Query coverage	Max identity	SSH-C	SSH-NC	SSH-S	SSH-A	SO	AO	N
Pathogen detection	Recognition	**C-type lectin 1**	blastx	ABA54612.1	C-type lectin 1	*Fenneropenaeus chinensis*	5E-03	0.44	0.21							x
			tblastx	DQ871245.1	C-type lectin	*Litopenaeus vannamei*	8E-09	0.27	0.48							
		**C-type lectin 2**	blastx	ACR56805.1	C-type lectin	*Fenneropenaeus merguiensis*	1E-08	0.39	0.30				x	x		x
			tblastx	CP000576.1	Prochlorococcus marinus str. MIT 9301	*Prochlorococcus marinus*	9E-05	0.12	0.50							
		**C-type lectin 3**	blastx	ACC86854.1	C-type lectin-like domain-containing protein PtLP	*Portunus trituberculatus*	1E-09	0.74	0.27							x
			tblastx	EU477491.1	C-type lectin-like domain-containing protein PtLP	*Portunus trituberculatus*	4E-14	0.56	0.65							
		**Peroxinectin-like A**	blastx	XP_002435528.1	Peroxinectin. putative	*Ixodes scapularis*	8E-27	0.85	0.32	x						x
			tblastx	XM_002406272.1	Peroxinectin. putative	*Ixodes scapularis*	1E-41	0.76	0.36							
		**Peroxinectin-like B**	blastx	XP_002406316.1	Peroxinectin. putative	*Ixodes scapularis*	7E-23	0.70	0.38	x						
			tblastx	EU934306.1	TSA: AD-573 salivary peroxidase	*Anopheles darlingi*	6E-23	0.52	0.48							
	Transduction	**ECSIT**	blastx	BAI40012.1	Evolutionarily Conserved Signaling Intermediate in Toll pathways	*Marsupenaeus japonicus*	5E-43	0.58	0.59							x
			tblastx	AB491495.1	Evolutionarily Conserved Signaling Intermediate in Toll pathways	*Marsupenaeus japonicus*	3E-51	0.63	0.60							
		**MyD88-like**	blastx	XP_001658635.1	Myd88	*Aedes aegypti*	4E-08	0.50	0.29							x
			tblastx	XM_001658585.1	Myd88	*Aedes aegypti*	4E-07	0.41	0.27							
		**SOCS2-like**	blastx	BAI70368.1	suppressor of cytokine signaling-2 like	*Marsupenaeus japonicus*	9E-35	0.81	0.47							x
			tblastx	AB516427.1	suppressor of cytokine signaling-2 like	*Marsupenaeus japonicus*	2E-34	0.74	0.50							
Immune response	AMP	**ALF 1**	blastx	ABP73291.1	anti-lipopolysaccharide factor isoform 2	*Penaeus monodon*	2E-26	0.39	0.59							x
			tblastx	AB453738.1	MjALF2	*Marsupenaeus japonicus*	8E-30	0.40	0.58							
		**ALF 2**	blastx	BAH22585.1	anti-lipopolysaccharide factor 2	*Marsupenaeus japonicus*	2E-05	0.68	0.28	x						
			tblastx	AB453738.1	MjALF2	*Marsupenaeus japonicus*	8E-19	0.79	0.40							
		**Crustin 1**	blastx	ACU25385.1	Crustin 4	*Panulirus japonicus*	5E-22	0.43	0.55							x
			tblastx	FJ797417.1	Crustin 1 (PJC1)	*Panulirus japonicus*	7E-24	0.47	0.58							
		**Crustin 2**	blastx	ACU25385.1	Crustin 4	*Panulirus japonicus*	1E-10	0.44	0.48							x
			tblastx	FJ797420.1	Crustin 1 (PJC1)	*Panulirus japonicus*	7E-34	0.35	0.66							
		**Crustin 3**	blastx	ACU25382.1	Crustin 1	*Panulirus japonicus*	2E-28	0.35	0.65							x
			tblastx	FJ797417.1	Crustin 1 (PJC1)	*Panulirus japonicus*	6E-34	0.44	0.53							
		**I-type lysozyme**	blastx	ACZ63472.1	i-type lysozyme-like protein 2	*Penaeus monodon*	7E-41	0.70	0.67							x
			tblastx	GQ478704.1	i-type lysozyme-like protein 2	*Penaeus monodon*	1E-42	0.57	0.62							
	Serine proteases	**Masquerade-like A**	blastx	ABY64694.1	Masquerade-like protein	*Armadillidium vulgare*	2E-112	0.50	0.99	x						x
			tblastx	EU216755.1	Masquerade-like protein	*Armadillidium vulgare*	5E-134	0.50	0.99							
		**Masquerade-like B**	blastx	CAA72032.2	Masquerade-like protein	*Pacifastacus leniusculus*	2E-86	0.67	0.47	x					x	x
			tblastx	EU216755.1	Armadillidium vulgare masquerade-like protein	*Armadillidium vulgare*	1E-97	0.37	0.75							
	Serine protease inhibitors	**a2-macroglobulin A**	blastx	ABY64692.1	alpha-2-macroglobulin	*Armadillidium vulgare*	1E-119	0.99	1.00	x						x
			tblastx	EU216753.1	alpha-2-macroglobulin	*Armadillidium vulgare*	6E-152	1.00	1.00							
		**a2-macroglobulin B**	blastx	AAX24130.1	alpha-2-macroglobulin	*Penaeus monodon*	2E-06	0.28	0.54							x
			tblastx	DQ988330.2	alpha 2 macroglobulin	*Litopenaeus vannamei*	2E-81	0.54	0.57							
		**a2-macroglobulin C**	blastx	ABI79454.2	alpha 2 macroglobulin	*Litopenaeus vannamei*	6E-27	0.38	0.51					x		
			tblastx	AY826818.1	alpha-2-macroglobulin	*Penaeus monodon*	1E-12	0.35	0.52							
		**a2-macroglobulin D**	blastx	BAC99073.1	alpha2-macroglobulin	*Marsupenaeus japonicus*	1E-10	0.84	0.26							x
			tblastx	EF073268.2	alpha-2-macroglobulin	*Litopenaeus vannamei*	4E-35	0.36	0.44							
		**a2-macroglobulin E**	blastx	ABK60046.1	alpha-2-macroglobulin	*Macrobrachium rosenbergii*	5E-43	0.98	0.42	x						
			tblastx	EF073269.1	alpha-2-macroglobulin	*Macrobrachium rosenbergii*	6E-64	0.97	0.48							
	Regulation of granular secretion	**Cyclophylin G**	blastx	ADD18906.1	peptidyl-prolyl cis-trans isomerase	*Glossina morsitans morsitans*	1E-62	0.72	0.71				x			
			tblastx	EZ543483.1	TSA: Crepidula fornicata 3374.Cfedg	*Crepidula fornicata*	7E-74	0.67	0.70							
	RNAi	**Piwi**	blastx	XP_002155913.1	PREDICTED: similar to Cniwi	*Hydra magnipapillata*	2E-93	0.73	0.51		x				x	x
			tblastx	XM_002155877.1	PREDICTED: similar to Cniwi (LOC100201838)	*Hydra magnipapillata*	4E-105	0.73	0.64							
		**Argonaute-like**	blastx	NP_001181904.1	argonaute-2	*Sus scrofa*	6E-55	0.97	0.50				x			
			tblastx	XM_001638444.1	predicted protein (NEMVEDRAFT_v1g180719)	*Nematostella vectensis*	3E-56	0.84	0.47							
	Stress response	**Ferritin A**	blastx	ABY75225.1	Ferritin	*Macrobrachium rosenbergii*	4E-67	0.47	0.74	x				x	x	x
			tblastx	EU371046.1	Ferritin	*Macrobrachium rosenbergii*	4E-80	0.48	0.75							
		**Ferritin B**	blastx	ABY75225.1	Ferritin	*Macrobrachium rosenbergii*	2E-50	0.66	0.57						x	x
			tblastx	EU371046.1	Ferritin	*Macrobrachium rosenbergii*	2E-59	0.77	0.58							
		**Ferritin C**	blastx	ABY75225.1	Ferritin	*Macrobrachium rosenbergii*	3E-58	0.72	0.69							x
			tblastx	EU371046.1	Ferritin	*Macrobrachium rosenbergii*	4E-68	0.74	0.80							
		**BIP2**	blastx	XP_001687763.1	AGAP000189-PA [Anopheles gambiae str. PEST]	*Anopheles gambiae*	7E-52	0.60	0.46						x	x
			tblastx	XM_002428865.1	conserved hypothetical protein	*Pediculus humanus*	1E-59	0.51	0.57							
	Detoxification	**Peroxiredoxin A**	blastx	ACS91344.1	Peroxiredoxin	*Fenneropenaeus indicus*	3E-56	0.81	0.56					x		x
			tblastx	GQ161914.1	Peroxiredoxin	*Fenneropenaeus indicus*	1E-117	0.82	0.85							
		**Peroxiredoxin B**	blastx	ACF35639.1	Peroxiredoxin 6	*Eriocheir sinensis*	1E-79	0.68	0.63					x		x
			tblastx	EU626070.1	Peroxiredoxin 6		4E-95	0.68	0.65							
		**Peroxiredoxin C**	blastx	AAP93584.1	thioredoxin peroxidase	*Apis mellifera ligustica*	8E-78	0.76	0.78						x	
			tblastx	NM_001030437.1	Peroxiredoxin	*Xenopus tropicalis*	4E-92	0.77	0.76							
		**Peroxiredoxin-like D**	blastx	XP_970660.2	PREDICTED: similar to 1-Cys peroxiredoxin	*Tribolium castaneum*	5E-07	0.51	0.70					x		
			tblastx	XM_965567.2	PREDICTED: similar to 1-Cys peroxiredoxin	*Tribolium castaneum*	1E-09	0.59	0.66							
		**Thioredoxin A**	blastx	XP_001608075.1	Thioredoxin-like protein	*Nasonia vitripennis*	2E-73	0.88	0.60						x	x
			tblastx	XM_001608025.1	Thioredoxin-like protein	*Nasonia vitripennis*	2E-84	0.88	0.64							
		**Thioredoxin B**	blastx	XP_973267.1	PREDICTED similar to Thioredoxin domain-containing protein 14 homolog (LOC662051)	*Tribolium castaneum*	4E-58	0.96	0.53						x	x
			tblastx	XM_968174.1	PREDICTED similar to Thioredoxin domain-containing protein 14 homolog (LOC662051)	*Tribolium castaneum*	3E-63	0.91	0.60							
		**Glutathione peroxidase**	blastx	AAY66814.1	selenium dependent salivary glutathione peroxidase	*Ixodes scapularis*	3E-39	0.95	0.43							x
			tblastx	EU399681.1	Glutathione peroxidase	*Metapenaeus ensis*	5E-36	0.71	0.57							
		**Cu/Zn SOD**	blastx	ABU55006.1	Copper/zinc superoxide dismutase	*Macrobrachium rosenbergii*	1E-30	0.43	0.47	x						x
			tblastx	EU077527.1	Copper/zinc superoxide dismutase	*Macrobrachium rosenbergii*	9E-32	0.31	0.71							
		**cytMnSOD**	blastx	CAR85669.1	cytoplasmic manganese superoxide dismutase	*Cyanagraea praedator*	2E-102	0.68	0.66	x				x		x
			tblastx	FM242568.1	cytoplasmic manganese superoxide dismutase	*Cyanagraea praedator*	8E-116	0.68	0.73							
	Coagulation	**Transglutaminase B**	blastx	AAK69205.1	Transglutaminase	*Pacifastacus leniusculus*	3E-70	0.78	0.54	x						x
			tblastx	AF336805.1	Transglutaminase	*Pacifastacus leniusculus*	8E-84	0.78	0.60							
	Cellular differentiation	**Astakine**	blastx	ACI02322.1	astakine variant 2	*Penaeus monodon*	3E-11	0.64	0.52							x
			tblastx	EU980445.1	astakine variant 2	*Penaeus monodon*	7E-15	0.72	0.49							
		**Runt**	blastx	CAD44571.1	runt protein 1b	*Pacifastacus leniusculus*	2E-45	0.67	0.65						x	
			tblastx	AJ506096.1	Pacifastacus leniusculus mRNA for runt protein	*Pacifastacus leniusculus*	8E-73	0.65	0.82							
	Apoptosis	**AIF-like**	blastx	NP_001121885.1	apoptosis-inducing factor	*Danio rerio*	7E-28	0.54	0.43							x
			tblastx	NM_001128413.1	apoptosis-inducing factor	*Danio rerio*	9E-30	0.52	0.49							
	Autophagy	**ATG7**	blastx	XP_002600056.1	hypothetical protein BRAFLDRAFT_79689	*Branchiostoma floridae*	2E-40	0.88	0.52				x			
			tblastx	NM_001129922.1	ATG7 autophagy related 7 homolog	*Xenopus tropicalis*	5E-40	0.68	0.61							
		**ATG12**	blastx	ADO32996.1	Autophagy-like protein ATG12	*Biston betularia*	3E-33	0.50	0.52				x			
			tblastx	HM449861.1	Autophagy-like protein ATG12	*Biston betularia*	1E-38	0.47	0.53							
Other	Cytoskeleton	**Kinesin**	blastx	NP_999817.1	kinesin II	*Strongylocentrotus purpuratus*	3E-159	0.81	0.83					x		x
			tblastx	NM_214652.1	kinesin II	*Strongylocentrotus purpuratus*	0.0	0.82	84.00							

### Immune gene expression

**Figure 3:**
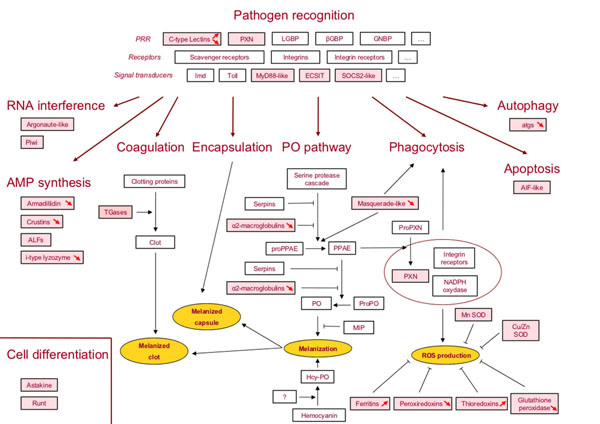
Pathway map for known crustacean immune functions: *Armadillidium vulgare* immune genes identified in this study were highlighted in pink boxes. The up and down arrows in gene boxes referred to significant up and down-regulation in symbiotic condition. AIF: Apoptosis inducing factor; ALF: Anti-lipopolysaccharide factor; LGBP: Lipopolysaccharide and β-glucan binding protein; βGBP: β-glucan binding protein; ECSIT: Evolutionary conserved intermediate in the Toll pathway; Hcy-PO: Hemocyanin with PO activity; MIP: Melanization Inhibitor Protein; PO: Phenoloxidase; PPAE: Prophenoloxidase activating enzyme; PXN: Peroxinectin; SOCS: Suppressor of cytokine signaling; SOD: Superoxide dismutase; TGase: Transglutaminase.

The expression of 46 candidate immune genes (Table 4 and Additional File 1: Primer pairs used for RT-qPCR quantification) were quantified in whole animal, ovaries and immune tissues of symbiotic and asymbiotic *A. vulgare* females. Forty four genes were selected through the procedure described above and 2 other genes were selected from previous studies [44,45]. Twelve genes were selected from the SSH-C (11 unigenes) and SSH-NC (1 unigene) libraries in order to examine whether *Wolbachia* induce an immune activation as observed in a challenged condition. All the 46 selected immune genes can be placed in known crustacean immune pathways (Figure 3). We considered genes involved i) in pathogen recognition (receptors and associated signaling pathways), ii) in RNA interference (RNAi), coagulation, PO pathway, phagocytosis, apoptosis, and autophagy or iii) encoding antimicrobial peptides (AMPs) [46-50].

**Table 4:** Expression of the candidate genes involved in the *A. vulgare* immune response. Transcripts of genes were quantified by RT-qPCR and normalized with the expression of the L8 ribosomal protein (RbL8) and the Elongation Factor 2 (EF2). The ratio of expression between symbiotic and asymbiotic conditions was calculated for each sample (F=whole females; Ov=ovaries; IT=immune tissues, see text). Over-expression and under-expression in symbiotic samples were highlighted in light grey and in dark grey respectively (* p<0.05; ** p<0.001; - no measurable response).

			ratio symbiotic /asymbiotic		
	Biological functions	Genes	F	Ov	IT
Pathogen Detection	Recognition	C-type lectin 1	*1.19*	3.42**	*1.55*
		C-type lectin 2	**0.90**	0.30**	-
		C-type lectin 3	0.47*	-	*1.06*
		Peroxinectin-like A	**0.93**	**0.09**	*2.03*
		Peroxinectin-like B	**0.72**	**0.93**	*2.03*
					
	Transduction	ECSIT	*1.44*	**0.63**	*1.48*
		MyD88-like	**0.86**	**0.78**	*1.45*
		SOCS2-like	-	**0.72**	*1.44*
					
Immune response	AMP	ALF 1	**0.77**	**0.57**	**0.68**
		ALF 2	**0.90**	*2.50*	*1.42*
		Armadillidine	0.44**	**0.83**	**0.95**
		Crustin 1	**0.57**	-	-
		Crustin 2	**0.77**	**0.48**	-
		Crustin 3	0.50**	0.47**	-
		i-type lyzozyme	0.63**	**0.44**	*1.77*
					
	Serine proteases	Masquerade-like A	**0.41**	*1.30*	*1.18*
		Masquerade-like B	0.36*	**0.33**	-
					
	Serine protease inhibitors	α_2_-macroglobulin A	**0.95**	*1.03*	*1.05*
		α_2_-macroglobulin B	**0.80**	**0.83**	*1.21*
		α_2_-macroglobulin C	**0.68**	0.32**	**0.74**
		α_2_-macroglobulin D	**0.56**	*1.88*	*1.47*
		α_2_-macroglobulin E	*1.44*	*1.68*	*3.05*
					
	Regulation of granular secretion	Cyclophilin G	**0.94**	**0.74**	*1.31*
					
	RNAi	Piwi	**0.95**	**0.74**	-
		Argonaute-like	**0.98**	**0.62**	*1.31*
					
	Stress response/Detoxification	Ferritin A	**0.95**	2.32*	*1.71*
		Ferritin B	**0.79**	**0.67**	-
		Ferritin C	**0.84**	1.90**	*1.65*
		BIP2	**0.86**	**0.57**	*1.23*
		Peroxiredoxin A	**0.45**	**0.39**	*1.59*
		Peroxiredoxin B	**0.58**	0.44**	*1.05*
		Peroxiredoxin C	-	0.02**	-
		Peroxiredoxin-like D	**0.71**	*1.16*	**0.53**
		Thioredoxin A	*1.59*	1.91**	*2.13*
		Thioredoxin B	**0.57**	*1.17*	**0.73**
		Glutathione peroxidase	**0.82**	0.17**	*1.09*
		Cu/Zn SOD	**0.45**	**0.68**	*1.12*
		cytMn SOD	**0.65**	**0.77**	*1.66*
					
	Coagulation	Transglutaminase A	**0.75**	*2.67*	*1.95*
		Transglutaminase B	*1.33*	*1.99*	*1.77*
					
	Cellular differenciation	Astakine	**0.98**	**0.49**	*2.08*
		Runt	*1.40*	**0.83**	*1.69*
					
	Apoptosis	AIF-like	-	**0.59**	-
					
	Autophagy	atg7	**0.73**	0.53**	**0.59**
		atg12	**0.92**	0.27*	**0.69**
					
Other	Cytoskeleton	Kinesin	**0.94**	**0.34**	*1.35*
					
			*S* >*A*		**S < A**

In symbiotic conditions, expression of these genes showed a general trend to a down-regulation in whole animals (37/43) and ovaries (31/44). On the contrary, 30 genes among 37 are over-expressed in immune tissues (Table 4 and Additional File 5: Expression profiles of genes studied in whole animals, ovaries, and immune tissues of *A. vulgare*).

Significant differential expressions in whole animals and ovaries were recorded for 16 genes, 12 of them were down-regulated and 4 up-regulated (Table 4). No significant differential expression was detected in immune tissues. Three genes involved in pathogen recognition, the C-type lectin 1, C-type lectin 2, and the C-type lectin 3 genes were differentially expressed. The C-type lectin 1 was up-regulated in ovaries whereas the C-type lectin 2 was down-regulated in the same tissue. Finally, the C-type lectin 3 was down-regulated in the whole animals. Three genes encoding AMPs were down-regulated: The armadillidin and the i-type lyzozyme genes in whole animals and the crustin3 gene in both whole animals and ovaries. One serine protease gene, the masquerade-like B, was also under-expressed in whole animals. Three genes involved in detoxification, the peroxiredoxin A and C and glutathione peroxidase, were down-regulated in ovaries whereas the thioredoxin A was up-regulated in the same tissue. In the autophagy pathway, two genes, atg7 and atg12, were under-expressed in ovaries. Among genes involved in stress response, the ferritin A and C genes were over-expressed in ovaries.

## Discussion

The different EST libraries generated in this study constitute the first reference transcriptome ever obtained in the Isopoda group [51]. Among crustaceans, only the *Daphnia pulex* (Branchiopoda, Cladocera) genome was recently published [52] and some EST libraries were constructed from a shrimp, a crayfish, and a porcelain crab (Malacostraca, Decapoda) [53-57]. Another EST database was obtained in the marine isopod *Limnoria quadripunctata*, but it concerned only the hepatopancreas [58]. Thus, our result represents the eighth largest sequencing effort for any crustacean, behind the cladoceran *Da. pulex* and the decapods *Litopenaeus vannamei* and *Petrolisthes cinctipes*, and the sixth EST data set for any Malacostraca species [51,57]. Few *A. vulgare* unigenes present similarities with crustacean ESTs. This could be in part explained by the phylogenetic distance between isopods and the crustaceans from which EST libraries or genomics data are available. However, the overlapping between libraries was low, suggesting that the sequencing effort should be increased.

The present work allowed us to identify the first immune gene repertoire from a terrestrial crustacean. Indeed, until now large scale characterizations of immune genes in crustaceans have been based on only a few model organisms, such as shrimps, crayfishes or crabs. All immune genes identified in *A. vulgare* are involved in canonical immune pathways (Table 4 and Figure 3): i) **pathogen detection** including recognition molecules such as the lectins and peroxinectins (PXN) that are able to distinguish between self and non-self particles and signal transducers; ii) **immune cellular responses** including opsonization molecules (*e.g.*, PXN and masquerade-like proteins) inducing phagocytosis and cellular encapsulation; iii) **immune humoral responses** involving clotting and coagulation reactions, production of AMPs, generation of reactive oxygen species, detoxification processes, and the proPhenoloxidase (proPO) cascade; and iv) **other pathways** connected to immune responses such as antiviral immunity (RNA interference), programmed cell death (apoptosis and autophagy), and cell differentiation such as hematopoiesis [49,50,59,60].

Although 40 new genes all involved in immune pathways have been identified, several key genes were lacking (Figure 3). This can be explained by three non-exclusive hypotheses: The relatively low depth of the sequencing effort, the weak annotation (44%) due to divergence between isopods and the other Arthropoda clades, and the absence of some immune genes in isopods. For example, genes encoding important innate immune receptors, such as GNBPs or Toll, and their signal transducers Imd, Dorsal, Cactus, Relish were known in different crustacean species [47,49,61,62] but were not identified in *A. vulgare*. PO activity is detected in crustaceans, but isopods such as chelicerates seem to lack PO enzyme and the corresponding gene [11,58,63,64]. In the same way, the PGRP genes have never been identified in crustacean EST libraries nor in the brine shrimp genome [47], which suggests that these genes could be absent in this clade.

A growing number of studies showed that the immune system of *Wolbachia*-infected animals is modulated at the molecular level [17,18,22]. In *A. vulgare*, it has recently been shown that *Wolbachia* impact immune cellular processes [10,11,65]. We show here that *Wolbachia* symbiosis leads to a down-regulation of some *A. vulgare* immune genes. Indeed, among the candidate genes tested, 72% are down-regulated in whole females, 75% in ovaries and 19% in immune tissues. Among the 46 genes analyzed, no significant differential expression was detected in the immune tissues, whereas the expression of 16 of them was significantly disturbed when *Wolbachia* were present in whole animals and ovaries. The impacted genes are involved in biological functions such as stress response and detoxification, autophagy, AMP synthesis, pathogen recognition, and proteolytic cascades.

Several impacted genes are involved in oxidative stress response. The production of reactive oxygen species (ROS) is one of the first lines of defence against invading microbes. High concentrations of ROS create oxidative stresses, resulting in damage to lipids, nucleic acids, and proteins and reducing life span so that complex antioxidant defence systems have evolved to minimize damaging ROS. Our study shows a down-regulation of antioxidant enzymes only in the ovaries. This result agrees with those obtained in *Drosophila* S2 cell line infected by *Wolbachia *[66] and in *A. tabida - Wolbachia* symbiosis [24] but not with those from the *Ae. albopictus* Aa23 cell line [22]. In parallel, we show an up-regulation of the thioredoxin gene that could be a response to down-regulation of other genes encoding antioxidant proteins. An alternative hypothesis is that this last gene could be induced by *Wolbachia* to reduce apoptosis and accelerate multiplication of gonadic cells. Indeed, in mice, this electron donor protein reduces the process of oxidant molecules but also increases cell proliferation and the inhibition of apoptosis [67].

There was a significant over-expression of Ferritins A and C in symbiotic ovaries. Ferritins are important iron sequestration proteins and play a crucial role in the iron-withholding defence system [68]. The up-regulation of ferritin genes could be an active cellular reaction for starving *Wolbachia* of iron, which would lead to bacterial growth limitation. Besides, this over-expression could be the result of the under-expression of the detoxification enzymes (Peroxiredoxin B and C and Glutathione peroxidase). As intracellular free iron produces ROS by the Fenton reaction in presence of H_2_O_2_, iron sequestration could reduce ROS production and thus avoid deleterious effects in the cell. Regardless, this result contrasts with that obtained in *A. tabida*-*Wolbachia* system [24,69] where the ferritin genes were under-expressed in symbiotic condition. This down-regulation could be due to the dependence phenotype of *A. tabida* - *Wolbachia* association for the oocyte maturation, whereas our model is a facultative *Wolbachia* symbiosis that is not involved in host oogenesis.

Autophagy was initially reported as a bulk self-degradation mechanism for the turnover of proteins and organelles. Autophagy can be induced *via* PGRP-LE, which is essential in the innate bacterial recognition in *Drosophila* resistance against *Listeria monocytogenes *[70] suggesting that this biological process is involved in the innate immune response against intracellular bacteria, viruses, and parasites [70,71]. In our study, the atg7 and atg12 genes involved in autophagy were down-regulated in ovaries. Autophagy-associated genes were down-regulated also in *A. tabida-Wolbachia* and *S. oryzae-*SPE symbioses [24,25], which suggests that this process is critical in bacterial symbiosis. We may hypothesize that this down-regulation was an active strategy of *Wolbachia* to reduce their elimination by their host.

In *Wolbachia-*infected whole animals, three AMP genes were under-expressed (*i.e.*, armadillidin, crustin 3, and i-type lyzozyme). Armadillidin and crustin are two Gram-positive AMPs [44,72]. The impact of *Wolbachia* on AMP gene expression varies according to the host-symbiont association. In infected *D. simulans* and *Ae. albopictus *[73], and in the silkworm cell line [74], *Wolbachia* did not disturb AMP expression. On the contrary, attacin and diptericin genes were down-regulated in an infected *D. melanogaster* S2 cell line [66], whereas many AMP genes were up-regulated in the mosquitoes *Ae. aegypti* and *An. gambiae* transfected by the *w*MelPop strain [17-19]. In the *A. tabida-Wolbachia* association, the defensin, lyzozyme and hymenoptaecin genes were under-expressed [24] as well as the coleoptericin 1 gene in *S.oryzae-*SPE symbiosis [25,75]. In *A. vulgare*, the down-regulation of AMP genes could be related to the higher septicaemia found in *Wolbachia*-infected animals [10,11].

Two recognition molecules, the C-type lectins 1 and 2, were up and down-regulated, respectively, whereas gene expression of the C-type lectin 3 was not detected in ovaries. The C-type lectins are mainly carbohydrate binding proteins involved in pathogen recognition, opsonization and encapsulation response, and antiviral response [76,77]. It has been shown that these proteins are also involved in symbiont interactions: C-type lectins were required for the symbiont acquisition in scleractinian corals [78,79] and the marine nematode *Laxus oneistus *[80]. In *Ae. aegypti* and *An. gambiae* transfected with the pathogenic *Wolbachia* strain *w*MelPop, the C-type lectin genes were up-regulated [17,18]. In *A. vulgare*, expression of the three C-type lectin genes presents different patterns, probably due to specific functions of each protein.

Unlike what was observed in ovaries, the C-type lectin 3 gene expression was significantly down-regulated in immune tissues of symbiotic females, which could impact pathogen recognition ability of the host. In the same way, the serine protease masquerade-like B gene was down-regulated. This protein family is involved in several biological functions such as pattern recognition, opsonization, cell adhesion activity [81], and in antiviral responses [82]. In our system, the under-expression of this masquerade-like gene could potentially impair these functions.

In symbiotic ovaries, one kinesin-related gene was down-regulated. This pattern observed by RT-qPCR was also confirmed by *in silico* comparison between SSH-A vs. SO libraries. Indeed GO analysis highlighted vesicle transport and microtubule motor activity as the only functions over-represented in asymbiotic ovaries. These functions were mainly associated with kinesin protein family. In *D. melanogaster*, kinesin-1 has been reported to be involved in *w*Mel *Wolbachia* transport toward the posterior part of the oocyte [83]. In *A. vulgare*, the relation between kinesin and *Wolbachia* is still unknown. Nevertheless, the down-regulation observed in symbiotic ovaries might be a host response for limiting the movement of *Wolbachia* in oocytes. In the weevil *S. oryzae*, the primary endosymbiont SPE seems to stimulate vesicle trafficking, which emphasizes the importance of this process in host-symbiont interactions [25].

## **Conclusion**

Our study represents the first transcriptomics approach that aims at deciphering the *A. vulgare-Wolbachia* interactions and it established the first reference transcriptome for isopods. In *A. vulgare*, *Wolbachia* colonize not only the ovaries but also other tissues, particularly the immune cells [65,84]. Therefore, perturbation of the host immune gene expression could be a direct effect of the bacteria on immunity. In such a scenario, *Wolbachia* would not be a silent bacterium and could counteract the host immune system to survive and establish a long term association with the host. The quantification of immune-related gene expression revealed a global trend to gene under-expression in *Wolbachia-*infected whole animals and ovaries. Unexpected modulation of immune gene expression in ovaries could reflect a *Wolbachia* strategy to manipulate the crucial tissue for vertical transmission. Surprisingly, most of the immune genes (30/37) tend to be up-regulated in immune tissues. This general up-regulation could compensate the immune depressive effect of *Wolbachia* previously described in *A. vulgare *[10,11,65]. These results conflict with those observed in insect cell lines where *Wolbachia* down-regulated immune-related genes [66,85] but are congruent with those obtained in transfected *w*Melpop mosquitoes [17-19]. More work needs to be done to check whether this up-regulation confers host pathogen protection as observed in *Drosophila* and mosquitoes [14,15,17,19].

## Competing interests

The authors declare that they have no competing interests.

## Authors' contributions

FC performed the RT-qPCR experiments and analysis, the bioinformatics analysis, and drafted the manuscript. JHG participated in the design of experiments, prepared the libraries, and participated in the sequence analysis. DC participated in the design of experiments, carried out the EST data processing and analysis, and helped for statistical analysis of expression data. GM helped to design RT-qPCR experiments and reviewed the manuscript. FG and PW sequenced the libraries. PG, CBV and DB conceived and coordinated the study, participated in its design, and drafted the manuscript. All authors read and approved the final manuscript.

## Supplementary Material

Additional file 1 Primer pairs used for RT-qPCR quantification. Click here for file

Additional file 2 Unigenes differentially represented between symbiotic and asymbiotic ovaries. Click here for file

Additional file 3 Processes and functions over-represented in *A. vulgare* ovaries in response to *Wolbachia* infection, biological process levels 4 and 6. Click here for file

Additional file 4 Immune unigenes present in SO, AO, SSH-S, SSH-A, SSH-C, and SSH-NC libraries. Click here for file

Additional file 5 Expression profiles of genes studied in whole animals, ovaries, and immune tissues of *A. vulgare*. Gene transcripts were quantified by RT-qPCR and normalized with the expression of the ribosomal protein (RbL8) and the Elongation Factor 2 (EF2). Each bar represents the mean of three independent measurements with standard error. Click here for file
