# Supplementary material for: Feminizing Wolbachia: a transcriptomics approach with insights on the immune response genes in Armadillidium vulgare
Source: BMC Microbiol. 2012 Jan 18;12(Suppl 1):S1. doi: 10.1186/1471-2180-12-S1-S1 (PMC3287506; doi:10.1186/1471-2180-12-S1-S1)
Supplement: Additional file 1 — Primer pairs used for RT-qPCR quantification. [file 1471-2180-12-S1-S1-S1.pdf]

Gene Name	Accesion number	Forward primer	Reverse primer	Amplicon length (pb)
α_2-macroglobulin A	EU216753.1	ATTGTGGCTCGGGAAC	CTCTGGCTGAGTCTGGA	192
α_2-macroglobulin B	FQ905899	GTCAGCGAGACTTATACATTACC	GCAGCTAACGCTTCATCA	155
α_2-macroglobulin C	FQ885631	GATGGGGACGACAATAAAG	ATTCGTTTGGAGGTGGTA	187
α_2-macroglobulin D	FQ899211	TGCTGTAACAGGCAGT	GTGAACGAGTCCTCCG	262
α_2-macroglobulin E	FQ893750	GACACTGTAGTCGCCC	TCGTAAGGCACCCTGA	225
AIF-like	FQ898988	ATTCTCCAGTCTCCATCGG	GGCTATTCCAACCACCTTT	241
ALF 1	FQ895095	AGTTTATGGTCCGATGGTGA	GAAGTCCTTCGTGGCCT	186
ALF 2	FQ893962	CAGCCACAGAGACACT	ACTCCAACATTCAGAGGT	186
Armadillidin	AY644458	GGTGGAGGTGGTGGATTCAA	TTTAGATGATTATCCTCTGTAGCCG	150
Argonaute-like	FQ906629	GAAGATTGGAGGCGTGAA	CGATTGATGGGATTGAGGA	142
Astakine	FQ904075	AGAAAATGACTCCGAATCAG	GCTTCAGGTAAATGTATCGTT	200
ATG7	FQ892863	TCCTTCAAATTCTCCACAACA	TCTATCGCGTCCAAATCC	191
ATG12	FQ896457	CTTTGCTCCATCACCAGA	TTCATCCTCGCCTCAAAC	198
BIP2	FQ898092	CATTTCCCTCACAAAGTTCC	CCATTACCCTCTCATCACC	216
Cyclophylin G	EU216759.1	TAAAGGAGAAGGTTACAAGGG	GTTTGCCATTGAAAGCCA	177
Crustin 1	FQ901343	AAAGATCGCCACCAGT	TTTGTCACGTCCTGGG	224
Crustin 2	FQ904008	CCAGAGACCTCGACCC	TGATCCCAAACTACTACCGATA	208
Crustin 3	FQ899403	AAGCAGTCGATCCATCT	ACGGCGCTTTACAAAC	255
ECSIT	FQ905410	AATGGTTTGCTGTCCTCTTC	CCCGCAATAGTCTTTCTTGT	139
Elongation Factor 2	FQ896398	GTCAGATCATTCCCACAACA	TTTCCTCAAATACCACTCCAC	164
Ferritin A	FQ887056	AGAACGTGAACATGCC	CGCAACTGTCTCCCTT	191
Ferritin B	FQ889513	GGGCTTCAGGATATTCTTTCTC	ACTCTAGAAATAATGTGGCCAA	170
Ferritin C	FQ906089	AAGCAAGTAAACCAGTCCC	ATAGCCTCAACCTGTTCTTC	117
i-type lysozyme	FQ902565	TCCGTTCCTCATCTCGTAT	ACCATCTCCATTACAATCCTG	171
Kinesin1	FQ904911	AATAAGAACAAAGCAGAGTCGG	AGATACATCCATTTCAACCACC	104
C-type lectin 1	FQ900396	GGGAGCCTGTTACGAT	GCCATACAAAGCGGAG	205
C-type lectin 2	FQ892973	ATCGGCGGAAACTACA	CCCATCGCTACCTCCA	183
C-type lectin 3	FQ906953	GGCTCACACCTTGCTTATATC	TTCTTACAGGGTAACCATTGCC	143
Masquerade-like A	ABY64694.1	ACACTTTGCGTCACAAC	CCTCCATCACCGTAGC	209
Masquerade-like B	FQ900550	ACATCGTACATGCGAAAC	TCCACCCAGTGACTGAA	255
MyD88-like	FQ906745	TTGTGGATTCAGAAAGGAGG	GCTGTCTGTATATCTTCGTCA	181
Glutathione peroxidase	FQ900254	TACTGAGGTATGAGCAACGC	TGGACGGACGTATTTGACT	165
Peroxinectin-like A	FQ893953	TCCCTCGTTGGTCCAC	ACATGGGTAGCATCAGT	182
Peroxinectin-like B	FQ893964	ATTGGACGACACTCGG	AGATGGGCCAAACCAG	225
Peroxiredoxin A	FQ903144	TCTCACCTTGCATGGG	CTGGAACGCTTGTACC	246
Peroxiredoxin B	FQ899986	TCCCTTATCCTGCTACTACC	GAAATTGCTTCTTGGCTTCT	167
Peroxiredoxin C	FQ889661	GTCTTGGTGGTCTAGCTTATC	TATGAGAAACAATCCGCGAA	112

Peroxiredoxin-like D	FQ886901	GGGAGGTTAACTTTGTTTATACT	CCCATGAATCTCCAAGGAAAT	177
Piwi	FQ899145	ATATGATGATTGGGTATGATGCT	ATCCCTGAACACAACTACTG	229
SOCS2-like	FQ907205	GTGAGAACAACTTTACCCAAG	TTTCGTGTCAAGTCCTGTC	220
Cu/Zn SOD	FQ893604	AAAGTGTAGCTCGGCG	CCAGCGTTTCCAGTCT	261
Mn SOD	ABY64695.1	AACTGGTGCTATCGCC	ACCCAACTGCACTGGA	181
RbL8	FQ895433	AACTGGAGATAGAGGCAAA	CCACCAGCAGCAATTC	159
Runt	FQ891956	AAGTCGGGGATGGAAC	GGGAGGAGTCGTACTGA	185
Thioredoxin A	FQ899701	CCCAAATTCTGCCCAGTT	TTTCCGATTCATTTAGGCATTC	235
Thioredoxin B	FQ893083	AGACCCTGTTTAGATTCCAAAG	CGAGGTACTTCTCATGTTGTT	123
Transglutaminase A	EU216757	AGGGAGTCTTAGTTGGC	CGGGTCTACAAGGGAT	189
Transglutaminase B	FQ893748	AGACGTTGAACGTGTGC	AGTGTCCGAGACTCTCC	189
