# Supplementary material for: Feminizing Wolbachia: a transcriptomics approach with insights on the immune response genes in Armadillidium vulgare
Source: BMC Microbiol. 2012 Jan 18;12(Suppl 1):S1. doi: 10.1186/1471-2180-12-S1-S1 (PMC3287506; doi:10.1186/1471-2180-12-S1-S1)
Supplement: Additional file 2 — Unigenes differentially represented between symbiotic and asymbiotic ovaries. [file 1471-2180-12-S1-S1-S2.pdf]

Accession number of ESTs corresponding to over-represented unigenes	Rj	Accession number	Description	OS	OA	N	SSH-S	SSH-A	SSH-C	SSH-NC	Description	Length	mean Sim	GOs	Enzyme codes	InterProScan
FQ892049	5,7	NA	NA	0	27	0	0	0	0	3	activin atype iia	664	42.25%	F:transferase activity; F:transmembrane receptor protein serine/threonine kinase activity; P:transmembrane receptor protein serine/threonine kinase signaling pathway; C:integral to membrane; F:protein serine/threonine kinase activity; C:membrane; F:protein kinase activity; F:transforming growth factor beta receptor activity; F:nucleotide binding; P:protein amino acid phosphorylation; F:ATP binding; F:kinase activity; F:receptor activity; F:metal ion binding; F:magnesium ion binding; F:manganese ion binding; F:receptor signaling protein serine/threonine kinase activity	NA	noIPR
FQ887701	2,5	NA	NA	6	0	7	0	0	0	0	---NA---	765			NA	no IPS match
FQ890062	2,1	NA	NA	0	10	8	0	0	0	0	---NA---	873	50.6%		NA	no IPS match
FQ892189	2,1	NA	NA	0	10	0	0	0	0	0	cation-transporting atpase	634	50.0%		NA	noIPR
FQ886179	2,08	B0X7W3|B0X7W3_CULQU	Putative uncharacterized protein - Culex quinquefasciatus (Southern house mosquito)	5	0	1	0	0	0	0	set and mynd domain-containing	1092	50.0%	C:nucleus	NA	noIPR
FQ886823	2,08	Q7ZXG5|Q7ZXG5_XENLA	3081 protein - Xenopus laevis (African clawe	5,00	0,00E+00	0	0	0,00	0,00	0,00E+00	ubiquitinisoform cra_a	725	98.9%		NA	no IPS match
