# Supplementary material for: Feminizing Wolbachia: a transcriptomics approach with insights on the immune response genes in Armadillidium vulgare
Source: BMC Microbiol. 2012 Jan 18;12(Suppl 1):S1. doi: 10.1186/1471-2180-12-S1-S1 (PMC3287506; doi:10.1186/1471-2180-12-S1-S1)
Supplement: Additional file 3 — Processes and functions over-represented in A. vulgare ovaries in response to Wolbachia infection, biological process levels 4 and 6. [file 1471-2180-12-S1-S1-S3.pdf]

	Biological process	GO accession	A	S	A/S
**AO ~ SO**	mitotic sister chromatid segregation	GO:0000070	0,05	0,09	**0,56**
**level 4**	meiotic spindle organization and biogenesis	GO:0000212	0,02	0,02	**1,00**
**(n= 217)**	sister chromatid segregation	GO:0000819	0,05	0,09	**0,56**
	regulation of action potential	GO:0001508	0,00	0,02	**0,00**
	response to hypoxia	GO:0001666	0,04	0,05	**0,80**
	blastocyst development	GO:0001824	0,04	0,07	**0,57**
	regulation of systemic arterial blood pressure by hormone	GO:0001990	0,11	0,14	**0,79**
	leukocyte mediated immunity	GO:0002443	0,02	0,02	**1,00**
	regulation of immune effector process	GO:0002697	0,04	0,14	**0,29**
	aldehyde metabolic process	GO:0006081	0,07	0,11	**0,64**
	electron transport	GO:0006118	3,82	6,14	**0,62**
	vitamin metabolic process	GO:0006766	0,14	0,21	**0,67**
	oxygen and reactive oxygen species metabolic process	GO:0006800	0,00	0,02	**0,00**
	smooth muscle contraction	GO:0006939	0,02	0,02	**1,00**
	humoral immune response	GO:0006959	0,11	0,14	**0,79**
	mitotic chromosome condensation	GO:0007076	0,02	0,05	**0,40**
	neurotransmitter secretion	GO:0007269	0,04	0,05	**0,80**
	spermatid development	GO:0007286	0,07	0,11	**0,64**
	eggshell chorion formation	GO:0007306	0,04	0,05	**0,80**
	neuroblast proliferation	GO:0007405	0,02	0,02	**1,00**
	sex determination	GO:0007530	0,09	0,14	**0,64**
	blood coagulation	GO:0007596	0,04	0,05	**0,80**
	hemostasis	GO:0007599	0,04	0,05	**0,80**
	regulation of heart contraction	GO:0008016	0,00	0,02	**0,00**
	axon ensheathment	GO:0008366	0,00	0,02	**0,00**
	response to virus	GO:0009615	0,02	0,02	**1,00**
	response to toxin	GO:0009636	0,00	0,05	**0,00**
	alkaloid metabolic process	GO:0009820	0,02	0,02	**1,00**
	carbon utilization by fixation of carbon dioxide	GO:0015977	0,05	0,09	**0,56**
	energy derivation by oxidation of organic compounds	GO:0015980	0,41	1,70	**0,24**
	macroautophagy	GO:0016236	0,02	0,05	**0,40**
	somatic diversification of immunoglobulins	GO:0016445	0,02	0,02	**1,00**
	viral genome replication	GO:0019079	0,02	0,02	**1,00**
	regulation of lipid metabolic process	GO:0019216	0,02	0,02	**1,00**
	telencephalon development	GO:0021537	0,02	0,02	**1,00**
	eggshell formation	GO:0030703	0,04	0,05	**0,80**
	oocyte fate determination	GO:0030716	0,02	0,02	**1,00**
	hindbrain development	GO:0030902	0,02	0,02	**1,00**
	reproductive behavior in a multicellular organism	GO:0033057	0,04	0,05	**0,80**
	regulation of membrane potential	GO:0042391	0,00	0,02	**0,00**
	response to drug	GO:0042493	0,07	0,09	**0,78**
	nitrogen compound biosynthetic process	GO:0044271	0,16	0,30	**0,53**
	innate immune response	GO:0045087	0,02	0,02	**1,00**
	homologous chromosome segregation	GO:0045143	0,02	0,02	**1,00**
	germ-line cyst encapsulation	GO:0048138	0,02	0,02	**1,00**
	vesicle docking	GO:0048278	0,04	0,05	**0,80**
	positive regulation of response to stimulus	GO:0048584	0,05	0,11	**0,45**
	regulation of immune response	GO:0050776	0,04	0,14	**0,29**
	negative regulation of multicellular organismal process	GO:0051241	0,04	0,05	**0,80**
	regulation of cell motility	GO:0051270	0,00	0,05	**0,00**
	establishment of chromosome localization	GO:0051303	0,00	0,02	**0,00**
	metaphase plate congression	GO:0051310	0,00	0,02	**0,00**

	meiotic metaphase plate congression	GO:0051311	0,00	0,02	**0,00**
	establishment of vesicle localization	GO:0051650	0,04	0,07	**0,57**
**AO ~ SO level 6 (n= 220)**	mitochondrial genome maintenance	GO:0000002	0,02	0,02	**1,00**
	regulation of cyclin-dependent protein kinase activity	GO:0000079	0,04	0,09	**0,44**
	G2 phase of mitotic cell cycle	GO:0000085	0,02	0,05	**0,40**
	sulfur amino acid biosynthetic process	GO:0000097	0,04	0,07	**0,57**
	sulfate assimilation	GO:0000103	0,02	0,02	**1,00**
	achiasmate meiosis I	GO:0000705	0,00	0,02	**0,00**
	long-chain fatty acid metabolic process	GO:0001676	0,02	0,02	**1,00**
	in utero embryonic development	GO:0001701	0,05	0,07	**0,71**
	somitogenesis	GO:0001756	0,00	0,02	**0,00**
	myeloid leukocyte differentiation	GO:0002573	0,00	0,02	**0,00**
	disaccharide metabolic process	GO:0005984	0,02	0,05	**0,40**
	chitin metabolic process	GO:0006030	0,00	0,02	**0,00**
	tricarboxylic acid cycle	GO:0006099	0,09	0,14	**0,64**
	pyrimidine nucleoside metabolic process	GO:0006213	0,02	0,02	**1,00**
	DNA fragmentation during apoptosis	GO:0006309	0,04	0,05	**0,80**
	protein amino acid glycosylation	GO:0006486	0,05	0,16	**0,31**
	protein amino acid lipidation	GO:0006497	0,02	0,02	**1,00**
	cysteine metabolic process	GO:0006534	0,02	0,05	**0,40**
	tryptophan metabolic process	GO:0006568	0,05	0,11	**0,45**
	indolalkylamine metabolic process	GO:0006586	0,05	0,11	**0,45**
	triacylglycerol metabolic process	GO:0006641	0,04	0,07	**0,57**
	phospholipid metabolic process	GO:0006644	0,12	0,18	**0,67**
	porphyrin biosynthetic process	GO:0006779	0,05	0,07	**0,71**
	vesicle coating	GO:0006901	0,00	0,05	**0,00**
	phagocytosis	GO:0006909	0,25	0,34	**0,74**
	phagocytosis. engulfment	GO:0006911	0,25	0,34	**0,74**
	mitotic centrosome separation	GO:0007100	0,04	0,05	**0,80**
	Notch signaling pathway	GO:0007219	0,02	0,02	**1,00**
	oocyte dorsal/ventral axis determination	GO:0007310	0,04	0,07	**0,57**
	sensory perception of sound	GO:0007605	0,02	0,02	**1,00**
	Toll signaling pathway	GO:0008063	0,02	0,02	**1,00**
	amino acid biosynthetic process	GO:0008652	0,12	0,30	**0,40**
	phospholipid biosynthetic process	GO:0008654	0,05	0,09	**0,56**
	aerobic respiration	GO:0009060	0,34	1,68	**0,20**
	glutamine family amino acid metabolic process	GO:0009064	0,09	0,14	**0,64**
	aromatic amino acid family biosynthetic process	GO:0009073	0,02	0,05	**0,40**
	histidine family amino acid metabolic process	GO:0009075	0,00	0,02	**0,00**
	coenzyme catabolic process	GO:0009109	0,09	0,14	**0,64**
	ribonucleoside metabolic process	GO:0009119	0,05	0,07	**0,71**
	nucleoside monophosphate biosynthetic process	GO:0009124	0,05	0,11	**0,45**
	oligosaccharide biosynthetic process	GO:0009312	0,00	0,05	**0,00**
	response to gamma radiation	GO:0010332	0,00	0,05	**0,00**
	synaptic vesicle budding	GO:0016185	0,00	0,05	**0,00**
	benzoate metabolic process	GO:0018874	0,05	0,07	**0,71**
	hexose metabolic process	GO:0019318	0,21	0,28	**0,75**
	pyridine nucleotide metabolic process	GO:0019362	0,05	0,09	**0,56**
	T cell differentiation	GO:0030217	0,00	0,05	**0,00**
	chromosome condensation	GO:0030261	0,02	0,07	**0,29**
	apoptotic nuclear changes	GO:0030262	0,04	0,07	**0,57**
	regulation of protein stability	GO:0031647	0,02	0,09	**0,22**

tetrapyrrole biosynthetic process	GO:0033014	0,05	0,07	**0,71**
eye photoreceptor cell development	GO:0042462	0,02	0,02	**1,00**
organelle ATP synthesis coupled electron transport	GO:0042775	3,04	5,36	**0,57**
positive regulation of apoptosis	GO:0043065	0,11	0,18	**0,61**
biopolymer methylation	GO:0043414	0,09	0,14	**0,64**
regulation of kinase activity	GO:0043549	0,18	0,28	**0,64**
cellular protein complex assembly	GO:0043623	0,05	0,21	**0,24**
dicarboxylic acid metabolic process	GO:0043648	0,04	0,16	**0,25**
cellular carbohydrate catabolic process	GO:0044275	0,16	0,23	**0,70**
indolalkylamine biosynthetic process	GO:0046219	0,02	0,05	**0,40**
monosaccharide catabolic process	GO:0046365	0,11	0,23	**0,48**
acylglycerol biosynthetic process	GO:0046463	0,00	0,07	**0,00**
retinal cell programmed cell death	GO:0046666	0,02	0,02	**1,00**
regulation of compound eye retinal cell programmed cell death	GO:0046669	0,02	0,02	**1,00**
negative regulation of retinal cell programmed cell death	GO:0046671	0,02	0,02	**1,00**
lipid phosphorylation	GO:0046834	0,00	0,09	**0,00**
positive regulation of lipid biosynthetic process	GO:0046889	0,02	0,02	**1,00**
synaptic vesicle endocytosis	GO:0048488	0,02	0,05	**0,40**
positive regulation of transferase activity	GO:0051347	0,04	0,05	**0,80**
negative regulation of transferase activity	GO:0051348	0,00	0,02	**0,00**
oocyte nucleus localization during oocyte axis determination	GO:0051663	0,02	0,02	**1,00**
