# Supplementary material for: Feminizing Wolbachia: a transcriptomics approach with insights on the immune response genes in Armadillidium vulgare
Source: BMC Microbiol. 2012 Jan 18;12(Suppl 1):S1. doi: 10.1186/1471-2180-12-S1-S1 (PMC3287506; doi:10.1186/1471-2180-12-S1-S1)
Supplement: Additional file 4 — Immune unigenes present in SO, AO, SSH-S, SSH-A, SSH-C, and SSH-NC libraries. [file 1471-2180-12-S1-S1-S4.pdf]

Library	Number of immune unigenes	BLAST identification	EST accession number
**SSH-C**	31	Serine protease	FQ893761
		Serine protease	FQ893667
		Serine protease	FQ893623
		Serine protease	FQ893394
		Serine protease	FQ893914
		Serpin	FQ893681
		Serpin	FQ893573
		Serpin	FQ893895
		Serpin	FQ893888
		alpha-2 macroglobilin	FQ893810
		alpha-2 macroglobilin	FQ893384
		alpha-2 macroglobilin	FQ893750
		SOD	FQ893491
		SOD	FQ893604
		Thioredoxin	FQ893739
		Thioredoxin	FQ893583
		Ferritin	FQ893815
		Ovoperoxidase	FQ893638
		Salivary peroxidase	FQ893265
		Hemolymph proteinase	FQ893669
		Hemocyanin	FQ893601
		Masquerade	FQ893563
		Masquerade	FQ893636
		Cathepsin	FQ893968
		Cathepsin	FQ893917
		Cathepsin	FQ893627
		Coagulation	FQ893366
		Coagulation	FQ893759
		Transglutaminase	FQ893748
		Catalase	FQ893488
		Catalase	FQ893510
		ALF	FQ893962
**SSH-NC**	29	Cniwi	FQ894315
		Kallikrein	FQ894504
		Cathepsin	FQ894158
		Cathepsin	FQ894300
		Cathepsin	FQ894626
		Cathepsin	FQ894347
		Cathepsin	FQ894150
		Cathepsin	FQ894548
		Cathepsin	FQ894418
		Cathepsin	FQ894512
		Cathepsin	FQ894234
		Cathepsin	FQ894583
		Cathepsin	FQ894379
		Cathepsin	FQ894370
		Cathepsin	FQ894326
		Cathepsin	FQ894229
		Cathepsin	FQ894083
		Cathepsin	FQ894021
		Cathepsin	FQ893994
		Cathepsin	FQ893988
		Hemocyanin	FQ894523
		Hemocyanin	FQ894494
		Hemocyanin	FQ894164
		Hemocyanin	FQ894087
		Hemocyanin	FQ894168

		Hemocyanin	FQ894161
		Hemocyanin	FQ893990
		14-3-3 protein	FQ894275
		ppaF	FQ891505
**SSH-S**	1	Serine protease	FQ892721
**SSH-A**	6	Vasa	FQ892823
		C-type lectin	FQ892973
		Thioredoxin	FQ893083
		Thioredoxin	FQ893118
		ppaF	FQ892839
		Kallikrein	FQ893020
**OS**	15	Serine protease	FQ887028
		Serine protease	FQ885101
		Glutathione peroxidase	FQ887758
		Glutathione peroxidase	FQ886901
		Thioredoxin peroxidase	FQ884946
		Thioredoxin	FQ887737
		SOD	FQ887525
		alpha-2 macroglobilin	FQ885631
		Lectin	FQ887089
		Lectin	FQ885351
		Ferritin	FQ887056
		Kallikrein	FQ886276
		ppaF	FQ886787
		Vasa	FQ885102
		Calreticulin	FQ885078
**OA**	21	Serine protease	FQ890866
		Ferritin	FQ889498
		Thioredoxin	FQ891067
		Thioredoxin	FQ888463
		Thioredoxin	FQ889351
		Thioredoxin peroxidase	FQ889661
		Masquerade	FQ887977
		Cathepsin	FQ891314
		Cathepsin	FQ888267
		Cathepsin	FQ889870
		Lectin	FQ888287
		Lectin	FQ888908
		Runt	FQ891956
		Kallikrein	FQ890153
		Kallikrein	FQ890190
		Autophagy protein	FQ888699
		Vasa	FQ889550
		Piwi	FQ889702
		Ferritin	FQ889704
		Calreticulin	FQ889703
		Bip2	FQ892076
