# Supplementary figures and images for: Feminizing Wolbachia: a transcriptomics approach with insights on the immune response genes in Armadillidium vulgare
Source: BMC Microbiol. 2012 Jan 18;12(Suppl 1):S1. doi: 10.1186/1471-2180-12-S1-S1 (PMC3287506; doi:10.1186/1471-2180-12-S1-S1)

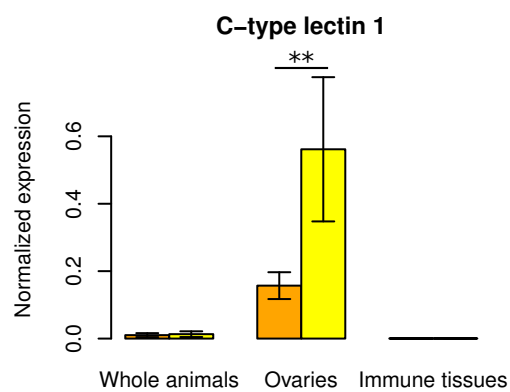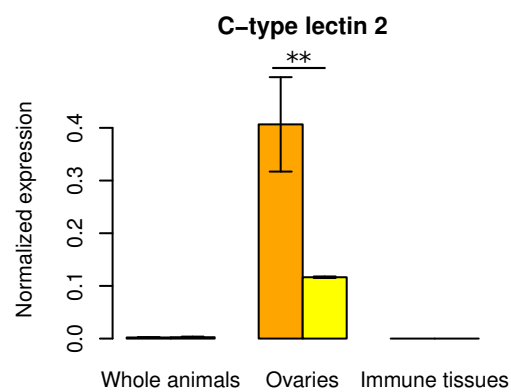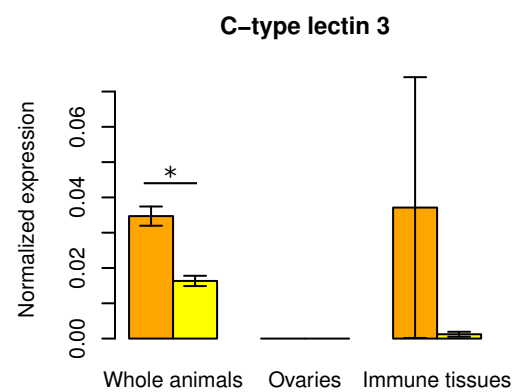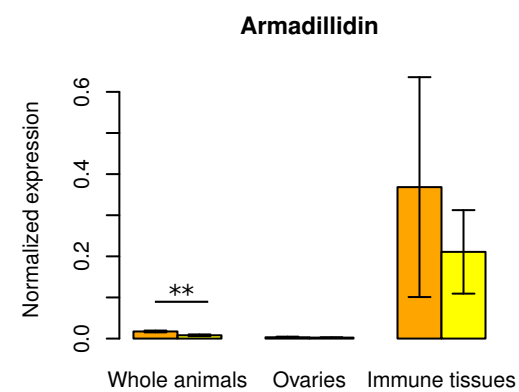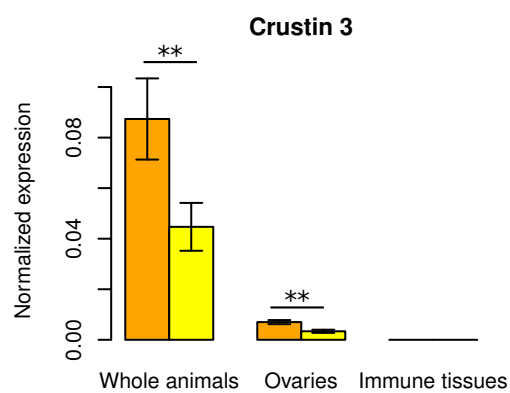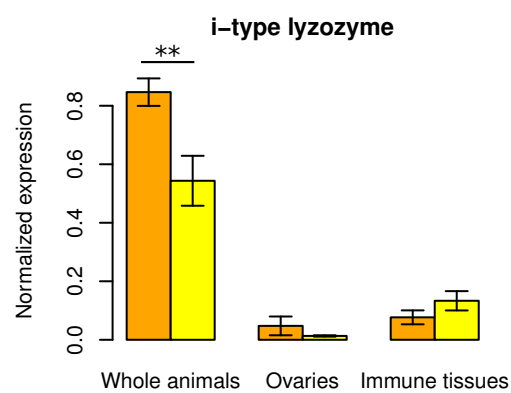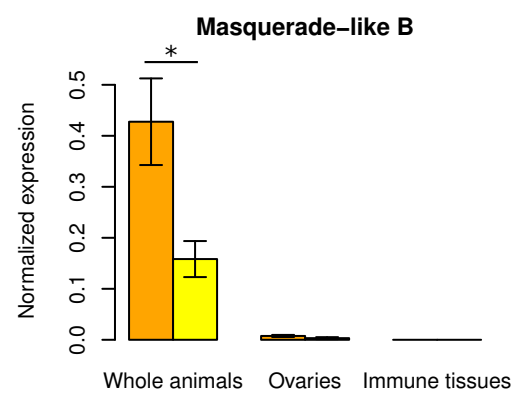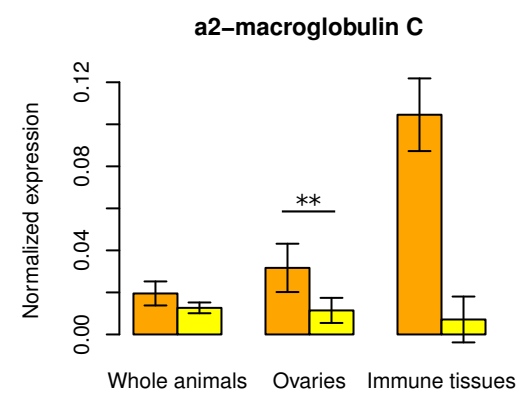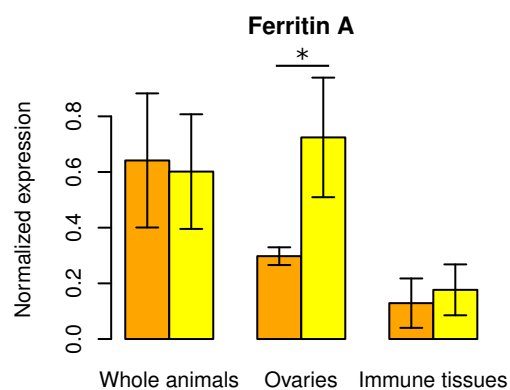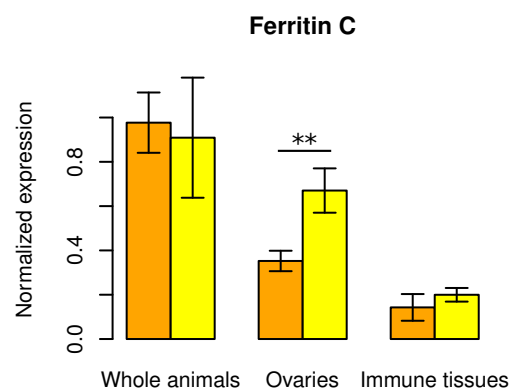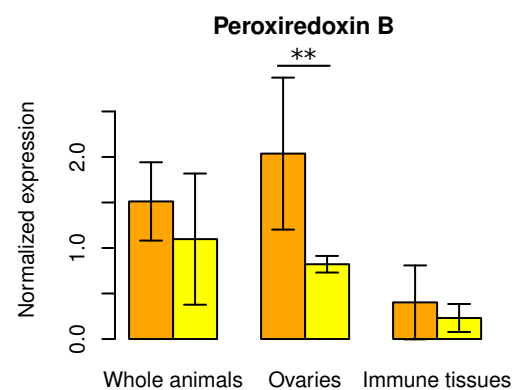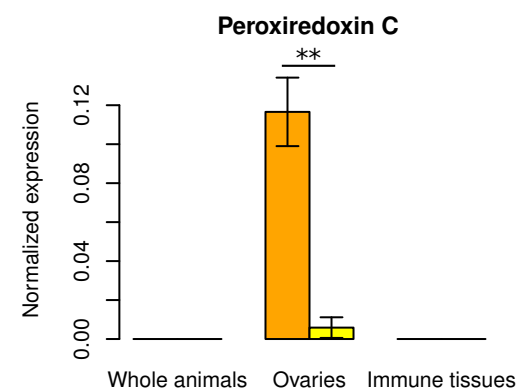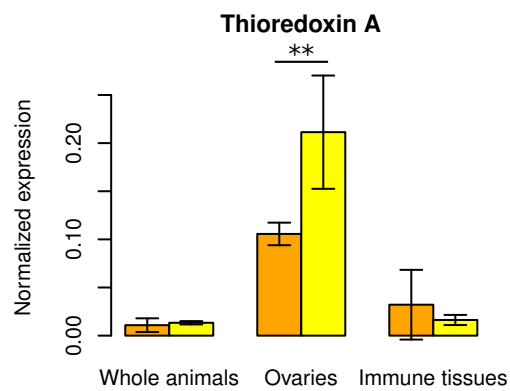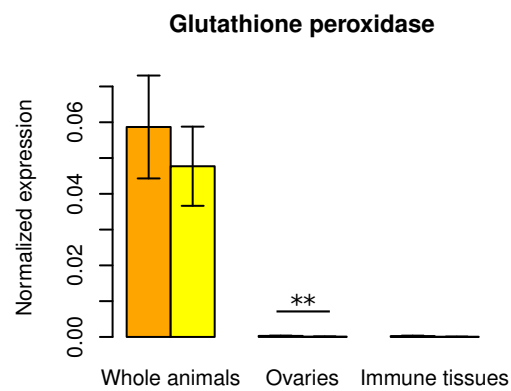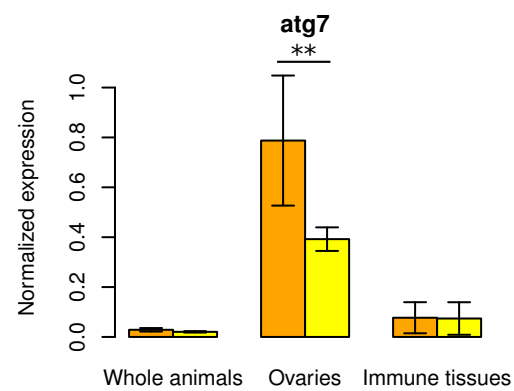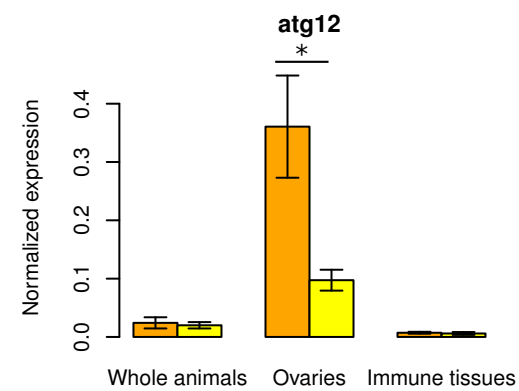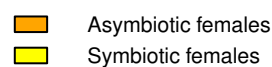

Supplement: Additional file 5 — Expression profiles of genes studied in whole animals, ovaries, and immune tissues of A. vulgare. Gene transcripts were quantified by RT-qPCR and normalized with the expression of the ribosomal protein (RbL8) and the Elongation Factor 2 (EF2). Each bar represents the mean of three independent measurements with standard error. [file 1471-2180-12-S1-S1-S5.pdf]
